# Calcium Signaling Derangement and Disease Development and Progression

**DOI:** 10.3390/biology10040291

**Published:** 2021-04-02

**Authors:** Vijay Sivaraman, Rob U. Onyenwoke

**Affiliations:** 1 Department of Biological and Biomedical Sciences, College of Health and Sciences, North Carolina Central University, Durham, NC 27707, USA; 2 Biomanufacturing Research Institute and Technology Enterprise (BRITE), North Carolina Central University, Room 2029, 1801 Fayetteville St., Durham, NC 27707, USA; 3 Department of Pharmaceutical Sciences, North Carolina Central University, Durham, NC 27707, USA

The importance of intracellular calcium (Ca^2+^) in regulating integral biological functions such as cell division, cell motility, autophagy, apoptosis and gene transcription through its capacity as a ubiquitous second messenger is clear. However, the delineation of the role of Ca^2+^ signaling within disease and disease progression is less defined.

Recent evidence demonstrates that Ca^2+^ signaling is dysfunctional in neurodegenerative disorders such as Alzheimer’s disease (AD), Parkinson’s disease (PD) and Huntington’s disease (HD) [1], with Ca^2+^ signaling dysregulation also associated with autoimmunity, asthma and even pulmonary cancer. In particular, the pulmonary consequences of deregulated Ca^2+^ homeostasis can include the development of pathology: airway inflammation, chronic obstructive pulmonary disease (COPD), acute lung injury (ALI), acute respiratory disease syndrome (ARDS) and lung cancer [2]. However, little information exists about the role intracellular Ca^2+^ concentration fluxes play in these pulmonary diseases. Herein, we will briefly highlight our current understanding of the part that Ca^2+^-signaling abnormalities play within the context of pulmonary disease, with specific emphasis on the role of endoplasmic reticulum (ER)-resident Ca^2+^ entry and its potential as a therapeutic target for treating pulmonary disease.

As previously mentioned, cellular Ca^2+^ is important for normal pulmonary physiology as well as regulating inflammatory responses against both environmental and pathogenic exposures [3]. Under basal states, intracellular Ca^2+^ levels are tightly regulated and maintained. However, when appropriately stimulated, intracellular Ca^2+^ release occurs. In particular, when Ca^2+^ is released inappropriately from the ER, significant depletion of ER-resident Ca^2+^ is associated with cellular apoptosis, pro-inflammatory responses and dysregulation of ciliary beat frequencies [4]. As such, modulation of Ca^2+^ signaling derangement has been shown to be associated with disease across the body. Of note, a recent emphasis has been placed upon electronic cigarette (also known as e-cigarette or E-cig) use or “vaping”, which presents but one vignette of Ca^2+^ signaling and disease.

The recent literature has presented a convincing association between the use of either the traditional combustible cigarette or e-cigarette use/vaping, and Ca^2+^ signaling and its dysregulation, which ultimately results in the cytotoxicity of pulmonary epithelia. In brief, we (and others) have utilized both in vitro and in vivo models to demonstrate that exposure to e-cigarette liquids (e-liquids, which are the actual products consumed during the vaping process) can elevate cytosolic Ca^2+^ levels and result in significant cytotoxicity and/or pathology [3,5,6,7,8]. Indeed, while one study has demonstrated that Ca^2+^ influx is diminished within the bronchial epithelia of traditional smokers, which is due to decreased ORAI3-dependent Ca^2+^ mobilization [3], studies have also shown that certain e-liquid flavor combinations specifically increase cytosolic Ca^2+^ levels within both the human bronchial epithelial cell line CALU-3 and primary-derived human bronchial epithelial cells. These latter observations correlate with increased cytotoxicity [5]. In addition, when using e-liquids previously demonstrated to significantly increase intracellular Ca^2+^ levels, increased levels of the inflammatory cytokine IL-6 as well as apoptosis and cell death were observed in both CALU-3 cells and a 2nd human epithelial cell line, A549 [6]. More recently, using an established method to deliver vaped e-liquids, we evaluated the effects of vaping upon pulmonary e-cigarette exposure in vivo using a murine model. Our results demonstrated a role for increased ER-resident Ca^2+^ release and the clear exacerbation of pulmonary disease when using either a bacterial or viral challenge, which was related to a prior acute vape exposure. These data strongly suggest that vaping may dysregulate intracellular Ca^2+^ stores, thus contributing to increased pathogenesis and morbidity after a pulmonary pathogen challenge. We have further utilized pharmacology, that is, small molecule treatments, to strengthen the association between a dual exposure, i.e., bacterial or viral challenge with prior vaping exposure, and Ca^2+^-mediated pathology. Finally, vaping has been theorized to induce the polarization of M0 macrophages to pro-inflammatory M1 macrophages, which is a process documented to be mediated by increased intracellular Ca^2+^ levels [7,9]. Thus, vaping may trigger macrophage polarization and lead to the accumulation of populations of inflammatory macrophages within the lung, which would increase pulmonary inflammation. This is another pro-inflammatory mechanism that we are currently investigating in vivo.

Because Ca^2+^ signaling is intrinsically important to general biological homeostasis, therapeutics cannot be designed to generally blunt signaling in the presence of a stimulating factor. This former description would be more akin to using a proverbial “hammer” to treat an ailment. Indeed, specific and targeted approaches must be pursued to modulate Ca^2+^ signaling and to diminish inflammation when warranted. However, such an approach does provide a level of complexity to studies involving Ca^2+^-dependent pathologies, thus requiring new approaches and innovative and, undoubtedly, intensive studies to develop the appropriate “rheostat” for therapeutic design. 

Thus, this special issue on “Calcium Signaling Derangement and Disease Development and Progression” addresses the complex set of circumstances that result when such an intrinsically important signaling cascade such as Ca^2+^ signaling is dysregulated and the resultant pathology that can result. The contained works will bring to light the new cutting edge and experimental results that will drive future study in this area, which will elucidate the framework of Ca^2+^ signaling and how its dysregulation can result in disease. Further, this issue is meant to highlight potential novel therapeutic applications and/or directions to ameliorate Ca^2+^ dysregulation, and thus disease, which will require interdisciplinary studies in the areas of genetics, toxicology, physiology and beyond.
